# Organ Donation: An Act of Individual Generosity and Civic Solidarity

**DOI:** 10.3389/ti.2025.16062

**Published:** 2026-01-08

**Authors:** Thierry Berney

**Affiliations:** Editor-in-Chief, Transplant International

**Keywords:** allocation systems, communication, information, organ donation, promotion of organ donation

When the editorial board received a few days ago the letter written by Reg Green [1], it only took the time to read it to make the decision to publish it in this issue of Transplant International. The letter reminds us of the story of a young boy from the United States, Nicholas Green, who was fatally shot during a robbery while on a family vacation in Italy. In spite of the tragedy that brutally hit the family, they rapidly made the decision to donate Nicholas organs, which allowed to perform transplants in a foreign country in 7 individuals battling illness.

Although this occurred 31 years ago, in the times of my surgical residency, I remember quite vividly this tragic event, the response the Green family decided to oppose to the immense grief and sense of injustice to which they had brutally been confronted and the enormous media coverage it received.

The “Nicholas effect” that ensued is not an overstatement. This act of generosity and solidarity had such a huge impact on the Italian society, that the rate of organ donation in the burgeoning Italian transplant program of the time was multiplied by 3 in the wake of the press coverage of these events [2]. A similar story occurred in the UK in 2017, when a young girl named Keira Ball was killed in a car accident. Her donated heart saved the life of Max Johnson, then 9 years of age. A successful campaign followed in the British press to support a change of the consent legislation from an “opt-in” to an “opt-out” policy, resulting in what is now known as the “Max and Keira law” [3, 4].

These two striking examples demonstrate how individual acts of generosity, not only have the power of bringing solace to a grieving family but can also bring about changes in the national consciousness of a population and in their appraisal of the importance of organ donation. It also shows the importance of communication and information campaigns to translate these acts into tangible results for individuals waiting for an organ.

According to the latest figures published by the Council of Europe, 167′000 organ transplants were performed worldwide in 2024, as reported to the Global Observatory on Organ Donation and Transplantation [5]. This impressive number pales in comparison to the 725′000 patients inscribed on waiting lists, not considering the unknown and, in all likelihood, masive numbers of individuals living with organ failure and no access to transplantation [6, 7].

Organ transplantation is a very particular medical activity. Not only does it shift the usual paradigm of a “contract” between a patient and a doctor, to a three-way relationship in which the organ donor is a crucial component, but it goes much beyond in integrating the notions of a “pool” of organ donors and a community of individuals waiting, sometimes for a very long time, for life-saving surgery. It implies a responsibility of states toward their populations to make every effort to maximize access to donor organs, of course in a total respect of ethical and legal principles [8, 9].

A European working group recently developed a conceptual framework for the planning and development of organ donation programs. They identified several domains of importance in 4 categories [10]. Structural and operational elements (national transplant organization, registries, structured transplant programs, …) are of course essential, but can only thrive thanks to actions -such as the training of hospital personel to approach potential donor families [4] and the building and maintainance of public support and trust [10]- that are based on communication and must be taken on a continuously ongoing basis. In this regard, it is a remarkable thing that, over the years, Reg Green has tirelessy pursued the endeavour of championing organ donation as a public health necessity. The message he is conveying in his letter [1] is that the acceptance of organ donation by the population should never be taken for granted and that communication and information campaigns never get the luxury of being put to rest even for a short while.

The “Spanish model” has been the epitome of a successful system resulting in the highest rate of organ donation and transplantation in the world [11]. Spain ticks every single box in the conceptual framework [10, 11], starting from a strong political support from the state, which has remarkably survived government changes, to strong structured infrastructures, an efficient program of donor identification [11–13], ethically acceptable incentives [14], thoughtful training of transplant coordinators to approach and speak with potential donor families [4] and regular information campaigns that begin in schools [8, 15]. “The success of the Spanish donation and transplantation program can most likely be attributed to policies that focus on trust and transparency. These include policies that support training opportunities for healthcare professionals that are focused on communication skills, family consultation, and consent, direct communication with the media, including educational programs for journalists, and round-the clock availability for consultation” [11]. It should come as no surprise that in this age of information and communication such policies should be the pillars of a successful organ donation model that boasts the highest rates of organ donation (54 per million population) and organ transplantation (132 per million population) from deceased donors in the world.

It has been reported and calculated that in a 25-year period (1987–2012), more than 500’000 persons had received an organ transplant in the USA, resulting in over 2 millon life-years saved [16]. In addition to saving lives, organ transplantation has the potential to improve quality of life [17, 18] and there is clear and sustained evidence that kidney transplantation is the most cost-effective therapeutic option for end-stage kidney disease, which actualy saves money to healthcare systems compared to dialysis [19]. Governments have therefore both a human and economic duty to maintain effective channels of information and promotion of organ donation. It could also be argued that citizens have a civic duty to consider becoming organ donors, thus participating in an act of national solidarity.

In Spain, in 2024, 6′310 patients received organs from 2′278 deceased donors, with 11′908 patients on an organ waiting list. Meanwhile, in Germany 3′618 patients received organs from 953 donors, with 13′945 on the waiting list. Depending on country of residence, the risk of being transplanted is 3–4 times higher than that of becoming an organ donor, and the risk of being on a waiting list for a life-saving organ that may never arrive is 5–15 times higher [5] ! A provocative question in campaigns aiming at increasing the number of potential organ donors -rather than asking if they would be willing to donate their organs after their death-could therefore be: “would you agree to become an organ recipient ?”, make the logical personal choice it implies and thus participate to what is a beautiful act of individual generosity and civic solidarity.
